# Improvement of Neuromuscular Synaptic Phenotypes without Enhanced Survival and Motor Function in Severe Spinal Muscular Atrophy Mice Selectively Rescued in Motor Neurons

**DOI:** 10.1371/journal.pone.0075866

**Published:** 2013-09-23

**Authors:** Ximena Paez-Colasante, Bonnie Seaberg, Tara L. Martinez, Lingling Kong, Charlotte J. Sumner, Mendell Rimer

**Affiliations:** 1 Department of Neuroscience and Experimental Therapeutics, College of Medicine, Texas A&M Health Science Center, Bryan, Texas, United States of America; 2 Texas A&M Institute for Neuroscience, Bryan, Texas, United States of America; 3 Department of Neurology, Johns Hopkins University, Baltimore, Maryland, United States of America; 4 Department of Neuroscience, Johns Hopkins University, Baltimore, Maryland, United States of America; University of Edinburgh, United Kingdom

## Abstract

In the inherited childhood neuromuscular disease spinal muscular atrophy (SMA), lower motor neuron death and severe muscle weakness result from the reduction of the ubiquitously expressed protein survival of motor neuron (SMN). Although SMA mice recapitulate many features of the human disease, it has remained unclear if their short lifespan and motor weakness are primarily due to cell-autonomous defects in motor neurons. Using *Hb9^Cre^* as a driver, we selectively raised SMN expression in motor neurons in conditional SMAΔ7 mice. Unlike a previous study that used choline acetyltransferase (*ChAT^Cre+^*) as a driver on the same mice, and another report that used *Hb9^Cre^* as a driver on a different line of conditional SMA mice, we found no improvement in survival, weight, motor behavior and presynaptic neurofilament accumulation. However, like in *ChAT^Cre+^* mice, we detected rescue of endplate size and mitigation of neuromuscular junction (NMJ) denervation status. The rescue of endplate size occurred in the absence of an increase in myofiber size, suggesting endplate size is determined by the motor neuron in these animals. Real time-PCR showed that the expression of spinal cord SMN transcript was sharply reduced in *Hb9^Cre+^* SMA mice relative to *ChAT^Cre+^* SMA mice. This suggests that our lack of overall phenotypic improvement is most likely due to an unexpectedly poor recombination efficiency driven by *Hb9^Cre^*. Nonetheless, the low levels of SMN were sufficient to rescue two NMJ structural parameters indicating that these motor neuron cell autonomous phenotypes are very sensitive to changes in motoneuronal SMN levels. Our results directly suggest that even those therapeutic interventions with very modest effects in raising SMN in motor neurons may provide mitigation of neuromuscular phenotypes in SMA patients.

## Introduction

Spinal muscular atrophy (SMA) is an often fatal, childhood inherited neuromuscular disease caused by low levels of a ubiquitous protein required for spliceosome assembly, survival of motor neuron (SMN). Reduction of SMN leads to loss of lower motor neurons, muscle denervation and atrophy. There is no current treatment for SMA. Humans harbor two genes encoding SMN, *SMN1* and *SMN2*. In SMA patients SMN production is drastically reduced by homozygous deletion/mutation of *SMN1*. In such patients SMN is solely derived from *SMN2*. *SMN2* is essentially identical to *SMN1* except for a C→T nucleotide change that causes exon 7 skipping in the splicing of ∼90% *SMN2* RNA, leading to the synthesis of an unstable, minimally functional protein (SMNΔ7). Only ∼10% of *SMN2* transcripts code for a full-length SMN protein and this reduced level is insufficient for motor neuron survival. *SMN2* can exist in multiple copies and SMA disease severity correlates inversely with *SMN2* copy number (reviewed in [1], [2]).

Mice only harbor one SMN gene, whose homozygous deletion (*Smn^−/−^*) is embryonic lethal. *Smn^−/−^* mice with two copies of *SMN2* (i.e. *Smn^−/−^; SMN2^+/+^*) die perinatally and show features resembling the most severe human disease [3]. Addition of a transgene encoding the cDNA for SMNΔ7 to these mice (i.e. *Smn^−/−^; SMN2^+/+^; SMNΔ7*) improves survival to 14 days on average. These animals, referred to as SMAΔ7 mice, still display a severe SMA phenotype. Thus, among other features, they demonstrate impaired motor behavior, inability to thrive and gain weight, and structural and functional defects in their neuromuscular junctions (NMJs) that include presynaptic accumulation of neurofilaments (NF), smaller and immature postsynaptic apparatus and diminished neurotransmission [4]–[8]. A selective vulnerability to denervation of specific, clinically relevant muscles has also been reported for these animals [9]. SMAΔ7 mice are the best characterized and the most widely used SMA model mice in preclinical trials.

**Figure 1:**
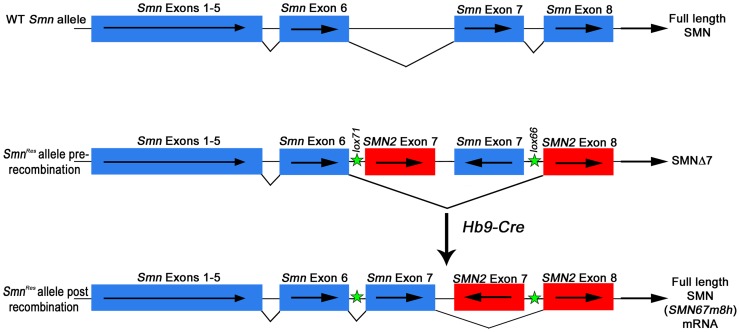
Schematic representation of the *Smn* WT allele and the *Smn^Res^* conditional hybrid mutant allele before and after Hb9-Cre recombination. Blue boxes represent mouse *Smn* exons. Red boxes represent human *SMN2* exons. Arrows within boxes display orientation relative to transcription start. Green stars show approximate location of *loxP* sites (*lox71, lox 66*) in *Smn^Res^*. Black lines connecting exons show splicing pattern for the predominant transcript encoded by each allele. Protein products are named to the right. *SMN67m8h* is the transcript encoded by the repaired *Smn^Res^* allele. Not drawn to scale.

Although SMA mice recapitulate many features of the human disease, it remains unclear if their short lifespan and SMA phenotype are primarily due to cell-autonomous defects in motor neurons. The general expectation in the field has been that selective SMN reduction in motor neurons should mimic the phenotype of the severe SMA model mice, and that conversely, selective restoration of SMN in motor neurons should dramatically rescue the phenotype of SMA model mice. These predictions have not been entirely borne out by the experiments. Thus, Olig2-Cre-driven conditional reduction of SMN in motor neuron precursors yields central and NMJ phenotypes in these animals that are present in the SMA mice, but the former animals have very mild SMA with a surprisingly long lifespan [10]. To conditionally restore SMN expression in motor neurons and other cells, mice with inducible mutant *Smn* alleles that can be reverted to functional, wild-type-like alleles after Cre recombination, were generated independently by two groups. These are the hybrid rescue allele, *Smn^Res^* [11] (Fig 1) and the *Smn^2B-neo^* allele [12]. Recently, *choline acetyltransferase-(ChAT)*-driven Cre was used to restore SMN expression in motor neurons of *Smn^Res/Res^* mice in the SMAΔ7 background [13], while *Hb9*-driven Cre was used to restore SMN expression in motor neurons of the *Smn^2B-neo/2B-neo^* SMA mice [14]. Mice rescued by both approaches showed measurable improvements in weight, motor behavior, central and neuromuscular phenotypes relative to diseased controls, but displayed a very modest increase in survival (8 and 5 days over diseased controls, respectively). These latter results contrast with the dramatic improvements, particularly in lifespan, produced by pan-neuronal expression of SMN in *Smn^−/−^; SMN2^+/+^* mice (210 days) [15], or in SMAΔ7 mice treated with CNS or peripherally-directed viral vectors expressing SMN (50–200 days) [16], [17], or CNS and peripherally-delivered oligonucleotides that correct the exon skipping defects in *SMN2* (>100 days) [18], [19].

Here we report on our attempt to restore SMN selectively in motor neurons of *Smn^Res/Res^* SMAΔ7 mice with the same *Hb9-Cre* driver used to rescue *Smn^2B-neo/2B-neo^* SMA mice. We found no improvement in survival, motor behavior, presynaptic NF accumulation and a marginal weight increase in our Hb9(Cre^+^)SMA mice relative to Hb9(Cre^−^)SMA mice. However, we detected rescue of endplate size and significant mitigation of NMJ denervation status in *Hb9^Cre+^* SMA mice. The rescue of endplate size was not a consequence of an increase in myofiber size. This pattern of mitigated peripheral structural phenotypes in *Hb9^Cre^* ^+^ SMA mice is similar to that in *Myf5^Cre^* ^+^ SMA mice [13], although the latter had restored SMN both in skeletal muscle and spinal cord, thus suggesting that this rescue in the *Myf5^Cre+^* SMA mice is due to neuronal and not muscle SMN restoration. Quantitative real time-polymerase chain reaction (qRT-PCR) showed that expression of the repaired *Smn^Res^* allele (*SMN67m8h*, Fig 1) was approximately 100-fold lower in spinal cord in our *Hb9^Cre+^* SMA mice than in the spinal cord from *ChAT^Cre+^* SMA mice. This suggests that our lack of overall phenotypic improvement is most likely due to an unexpectedly poor recombination efficiency driven by *Hb9^Cre^*. Nonetheless, our data show that specific structural NMJ phenotypes are very sensitive to small changes in SMN levels selectively in motor neurons and thus can be mitigated by very small increases in spinal cord SMN levels that otherwise fail at enhancing lifespan, weight and motor behavior. Although prior studies (e.g. [9], [20], [21]) have suggested that therapeutic interventions with very modest effects in raising SMN could potentially mitigate motor neuron-autonomous phenotypes in SMA patients, our results are the first to reach that conclusion directly based on an approach to selectively raise SMN levels in motor neurons.

## Materials and Methods

### Ethics Statement

Care and treatments of all animals followed the National Institutes of Health Guide for the Care and Use of Laboratory Animals, and were approved by the Institutional Animal Care and Use Committee (IACUC) of Texas A&M University under animal use protocol 2010-0258.

### Mice

Mice were housed in a vivarium at 25°C with a 12 h light/dark cycle, fed *ad libitum* and monitored daily for health. Human alleles are referred in the text with the standard convention of capital letters in *italics*. *Smn^Res^* carriers (*Smn^Res/+^; SMNΔ7^+/+^; SMN2^+/+^*, JAX stock #007951) and *Hb9^Cre^* carriers (*Hb9^Cre+/−^; Smn^+/−^*; *SMNΔ7^+/+^; SMN2^+/+^*, JAX stock # 007022) were initially acquired from the Jackson Labs, Bar Harbor, ME. The *Hb9^Cre^* allele in 007022 came from JAX stock# 006600, the same Hb9-Cre line used by DiDonato and colleagues to rescue *Smn^2B-Neo/2B-Neo^* mice [14], and that has been used by others in many developmental studies of motor neurons (e.g. [22], [23]). These lines were bred in our facility to generate *Hb9^Cre+/−^*; *Smn^Res/+^; SMNΔ7^+/+^; SMN2^+/+^* mice. For experiments, the latter were bred to *Smn^Res^* carriers. The genotype for the rescued animals, designated as Hb9(Cre^+^)SMA, was *Hb9^Cre+/−^*; *Smn^Res/Res^; SMNΔ7^+/+^; SMN2^+/+^*. The genotype for the disease animals, designated as Hb9(Cre^−^)SMA, was *Hb9^Cre−/−^*; *Smn^Res/Res^; SMNΔ7^+/+^; SMN2^+/+^*. Controls were *Smn^Res/+^* or *Smn^+/+^* mice derived from the same crosses.

The initial design of the study assumed that precise measures of lifespan, from birth until death, were essential to determine the effects of the selective raising of SMN in motor neurons of SMA mice. Thus, the approved animal use protocol for this study allowed death without euthanasia as an endpoint for the survival analysis. This accommodation turned out to be critical as the results showed that our experimental manipulation had no effect on survival. Had the differences in lifespan been more clear between our rescued and diseased animals, it would have been possible for us to establish a set of symptoms and criteria that would have allowed euthanasia before death without fear of changing or affecting the final results of the survival analysis. To minimize the number of SMA mice used in the study, animals that were followed for survival analysis were also used for longitudinal studies of weight progression and righting reflex. A separate set of control and SMA mice were used later to perform the hindlimb suspension test. This experiment was terminated at postnatal day (P) 12, before the SMA animals reached their mean lifespan of ∼14 days. NMJ phenotypes were analyzed in P9-P11 animals. Detailed descriptions of methods for all these experiments are provided below.

### Genotyping

Upon either death or weaning of the experimental progeny, tissue samples were obtained from tail, spinal cord, skeletal muscle, kidney, brain, heart and stomach. DNA was extracted and amplified by PCR using REDExtract-N-Amp Hot start according to manufacturer instructions (Sigma-Aldrich, St. Louis, MO). Primers: *Cre* was detected using the custom primers: Forward: 5′-CATTTGGGCCAGCTAAACAT-3′, Reverse: 5′-CCCGGCAAAACAGGTAGTTA-3′. The product was 454 bp. *Smn^−^* was detected with the following custom primers: Forward: 5′-CTTGGGTGGAGAGGCTATTC-3′, Reverse: 5′-AGGTGAGATGACAGGAGATC-3′. *Smn^+^* was detected with primers oIMR7033 and oIMR7034 (Jackson labs, Bar Harbor, ME). *SMN2* was detected with primers oIMR5065, oIMR5066, oIMR5067 (Jackson labs, Bar Harbor ME).

### Quantitative-real time-polymerase chain reaction (qRT-PCR)

Total RNA was extracted from P4 whole spinal cords and hindlimb skeletal muscle using Trizol reagent according to manufacturer instructions (Life Technologies, Grand Island, NY) and stored at -80°C until use. 1 µg of RNA per sample was reverse transcribed to cDNA using the High Capacity cDNA reverse transcription kit according to manufacturer instructions (Life Technologies, Grand Island, NY). Quantitative-RT-PCR for full length SMN transcripts encoded by the recombined *Smn^Res^* allele (*SMN67m8h*, Fig 1) was performed as described previously by Martinez et al. [13]. The 18S rRNA primers and probe were from Life Technologies, Grand Island, NY. Samples were set up in triplicate using a Taqman® Master mix (Life Technologies, Grand Island, NY) and run for 48 cycles in an ABI Prism 7900HT Fast Real-Time PCR System (Life Technologies, Grand Island, NY). The ΔΔCt method [24] was used to determine changes in mRNA transcript expression levels between groups.

### Survival analysis

Our experimental litters were monitored twice daily for potential mortalities. As soon as death of an animal occurred, lifespan was recorded and samples of the above-mentioned tissues were taken for genotyping. All animals assayed (n = 187) were grouped according to genotype and lifespan (days). Survival data was plotted on Kaplan-Meier survival curves using Prism5 (GraphPad Software, La Jolla, CA).

### Weight progression

On P3, pups were tattooed with Indian black ink for identification purposes and henceforth weighed every three days to determine weight changes over the time course of 21 days (until weaning for controls and death for mutants). All animals assayed (n = 150) were grouped according to genotype and mean weights for each time point were plotted using Prism5 (GraphPad Software, La Jolla, CA).

### Motor function

A righting reflex test was performed every three days on the same animals used for weight progression above. The test consisted of placing the pup in supine position on a flat surface and measuring the time it took to turn over onto an upright position, or to “right” itself, by having all four paws simultaneously on the flat surface. This measurement was taken in duplicate and averaged. All animals assayed (n = 150) were grouped according to genotype and mean time to “right” for each time point was scored according to the following scale, modeled after Monani and co-workers [10]: 0−4 s = 5, 5−9 s = 4, 10−14 s = 3, 15−20 s = 2, 21−49 s = 1, 50−>60 s = 0. A score of 0 was considered a failed test. Scores were plotted using Prism5 (GraphPad Software, La Jolla, CA). The hindlimb suspension (tube) test was performed as described by El-Khodor and colleagues [25]. Only latency time to fall (up to 60 s) was considered in the analysis.

### Neurofilament (NF) accumulation assay

NMJ staining was performed on whole mounts of P10-P11 tibialis anterior (TA) muscle. Pups were sacrificed with CO_2_, muscles were dissected, fixed in 4% paraformaldehyde (PFA) for ∼2 h at room temperature and washed extensively in PBS. To facilitate antibody penetration, forceps were used to break muscle into bundles that remained attached to one tendon. Such muscles were then permeabilized in methanol for 5 min at −20°C, and washed in PBS. Muscles were blocked for 1 h in PBS+0.2% BSA, 0.3% Triton X-100. To label NF's, samples were incubated overnight at room temperature on a rocker with 2H3 monoclonal antibody (1∶100, Developmental Studies Hybridoma Bank, Iowa City, IA). The following day, muscles were washed three times in PBS-T (PBS+0.1% Triton X-100) and then incubated for 2 h in rhodamine goat anti-mouse secondary antibody (1∶200, Jackson Immunoresearch, West Grove, PA) and fluorescein-conjugated α-bungarotoxin (BTX) (1∶1000, Life Technologies, Grand Island, NY) to label acetylcholine receptors (AChRs). After washing samples three times in PBS-T, muscle bundles were separated and mounted on microscope slides using VectaShield (Vector Laboratories, Burlingame, CA). Slides were observed under an epifluorescence microscope fitted with a Z-motorized stage (Nikon Eclipse E1000, Nikon, Tokyo, Japan) with a 100X, 1.3 NA oil objective. Images of NMJs were taken with a CoolSNAP fx CCD camera (Photometrics, Tucson, AZ) and captured with MetaMorph (Molecular Devices, Downingtown, PA). An observer, blind to genotype, initially classified NMJs as either NF accumulation positive (diseased) or negative (normal). NMJs were later grouped according to genotype for quantitative analysis.

### Innervation status

P9 triceps muscles were dissected, fixed in 4% PFA for 24 h and cryoprotected in sucrose at 4°C for another 24 h. Muscles were sliced in a cryostat into 40 µm longitudinal sections. Free-floating sections were blocked in PBS+10% normal goat serum for 1 h at room temperature. Sections were later incubated overnight in either an antibody to synaptophysin (1∶300, Life Technologies, Grand Island, NY) or in a 2H3 monoclonal antibody (1∶100, Developmental Studies Hybridoma Bank, Iowa City, IA). The following day all sections were washed in PBS-T and incubated for 2 h either in rhodamine goat anti-rabbit antibody (1∶1000, Jackson Immunoresearch, West Grove, PA) or in rhodamine goat anti-mouse secondary antibody (1∶200, Jackson Immunoresearch, West Grove, PA) respectively. Fluorescein-conjugated α-bungarotoxin (BTX) (1∶1000, Life Technologies, Grand Island, NY) was added to label acetylcholine receptors (AChRs). After washes in PBS-T, all free-floating sections were mounted on slides using VectaShield. A 40X, 1.3 NA oil objective was used to acquire wide field images of NMJs with MetaMorph Software. Endplates stained with synaptophysin were assessed blind to genotype for their innervation status by qualitatively classifying each junction into either fully-occupied (>75%), partially-occupied (25–75%) and unoccupied (<25%) depending on the relative proportion of synaptophysin staining versus the overall area of the AChR staining. NMJs stained for NF and BTX were assessed for status of innervation by categorizing them as innervated if there was any overlap between the two stains or denervated if NF was completely absent at the synaptic site.

### Endplate size

AChR area was calculated from the images of P9 TA whole mounts and from P9 triceps longitudinal sections using the MetaMorph software.

### Myofiber area and diameter

P9 TA muscles were dissected and flash frozen with OCT medium (tissue tek) in an isopentane/liquid N_2_ bath. 14 µm-thick cross-sections were cut in a cryostat and mounted on a slide. Sections were fixed in 1% PFA for 10 min and then washed three times in PBS-T for 5 min each. The cross sections were blocked in PBS+10% normal goat serum for 20 min at room temperature and then incubated overnight in a dystrophin antibody (1∶300, Abcam, Cambridge, MA). After three 15 min washes in PBS-T the following morning, the muscle sections were incubated at room temperature for 2 h with a fluorescein-conjugated goat anti-rabbit secondary antibody. After washes in PBS-T, slides were mounted using VectaShield. A 20X, 0.5 NA oil objective (Nikon) was used to acquire wide field images of myofibers with MetaMorph Software, taking as many images needed to encompass the entire area of a single whole muscle section avoiding overlapping myofibers. The dystrophin stained images were analyzed for myofiber area and diameter by first thresholding the calibrated image for dark objects and manually adjusting said threshold to fill the entire interior core surrounded by dystrophin staining of as many myofibers as possible per image. Next regions were drawn automatically around each individual fiber, and lastly the integrated morphometric analysis tool in MetaMorph was used to determine area and breath, which is the caliper width of the object perpendicular to the longest chord. This approach allows simultaneous accurate determination of myofiber size parameters in a large number of fibers. It is quicker than the traditional approach of measuring these parameters in single fibers at a time.

### Statistical analysis

Quantitative data are expressed as mean ± SEM. Values for number of animals and NMJs analyzed are given in the figure legends. Kaplan-Meier survival curves were generated and tested for statistical significance using the log-rank test with Prism5 GraphPad Software. Analysis of the Variance (ANOVA) was used to check for significance in weight and motor function data using SAS software (SAS Institute, Cary, NC). Student's t-test, computed either with Microsoft Excel (Microsoft Corporation, Seattle, WA) or Prism5 GraphPad Software, was used to probe for statistical significance in NF accumulation, innervation status and endplate size data. Significance was set at p≤0.05.

## Results

### Spinal cord-specific recombination of the *Smn^Res^* allele

The hybrid *Smn^Res^* allele has an inverted (i.e. 3′→5′), translationally silent mouse *Smn* exon 7 in the intron between human *SMN2* exon 7 and 8 (Fig 1). Mutant (*lox71/lox66*) *loxP* sites in opposite orientation were engineered upstream of *SMN2* exon 7 and downstream of the inverted *Smn* exon 7, respectively. In this form, this allele potentially codes for transcripts containing *Smn* exons 1–6 and *SMN2* exons 7 and 8. However, it was found that in this configuration *Smn^Res^* fails to encode transcripts for full-length SMN and only produces transcripts lacking *SMN2* exon 7 that code for SMNΔ7 protein (Fig 1). Hence *Smn^Res^* is functionally a null *Smn* allele [11]. Upon Cre recombination, *Smn* exon 7 5′→3′ orientation is irreversibly restored so that transcripts containing *Smn* exons 1–7 and *SMN2* exon 8 *(SMN67m8h)* are generated, which encode full length SMN (Fig 1). *Smn^Res/Res^* mice are embryonic lethal, however, when *Smn^Res^* replaces the *Smn* null allele in an SMAΔ7 background, the resulting *Smn^Res/Res^* mice display essentially the same SMA phenotype as standard SMAΔ7 mice [11]. Thus, these mice are “conditional” SMAΔ7 mice. Repairing the *Smn^Res^* allele using Cre-lines that express the recombinase ubiquitously from early development rescued the normal phenotype of the mice, validating the recombined, hybrid *Smn^Res^* allele as a WT-surrogate *Smn* allele [11].

To restore SMN expression selectively in motor neurons, we sought to drive the repair of the *Smn^Res^* allele with *Hb9*-Cre. The homeobox gene Hb9 is selectively expressed in spinal cord somatic and visceral motor neurons and V_x_ interneurons starting at embryonic (E) day 9.5 [26], [27]. In the *Hb9^Cre^* allele an IRES-Cre cassette was knocked-in into the first exon of the *Hb9* gene. Thus, Cre expression under *Hb9* control follows the expression pattern of endogenous *Hb9*. *Hb9^Cre^* is homozygous lethal thus only heterozygous animals survive past birth. The experimental crosses consisted of *Smn^Res^* carriers (*Smn^Res/+^; SMNΔ7^+/+^; SMN2^+/+^*, JAX stock #007951) bred to *Hb9^Cre+/−^*; *Smn^Res/+^; SMNΔ7^+/+^; SMN2^+/+^* mice. The genotype for the rescued animals, designated as Hb9(Cre^+^)SMA, was *Hb9^Cre+/−^*; *Smn^Res/Res^; SMNΔ7^+/+^; SMN2^+/+^*. The genotype for the disease animals, designated as Hb9(Cre^−^)SMA, was *Hb9^Cre−/−^*; *Smn^Res/Res^; SMNΔ7^+/+^; SMN2^+/+^*. Controls were *Smn^Res/+^* or *Smn^+/+^* mice derived from the same crosses. Their precise genotypes were: *Hb9^Cre?/−^*; *Smn^Res/+^; SMNΔ7^+/+^; SMN2^+/+^* and *Hb9^Cre?/−^*; *Smn^+/+^; SMNΔ7^+/+^; SMN2^+/+^*.

**Figure 2:**
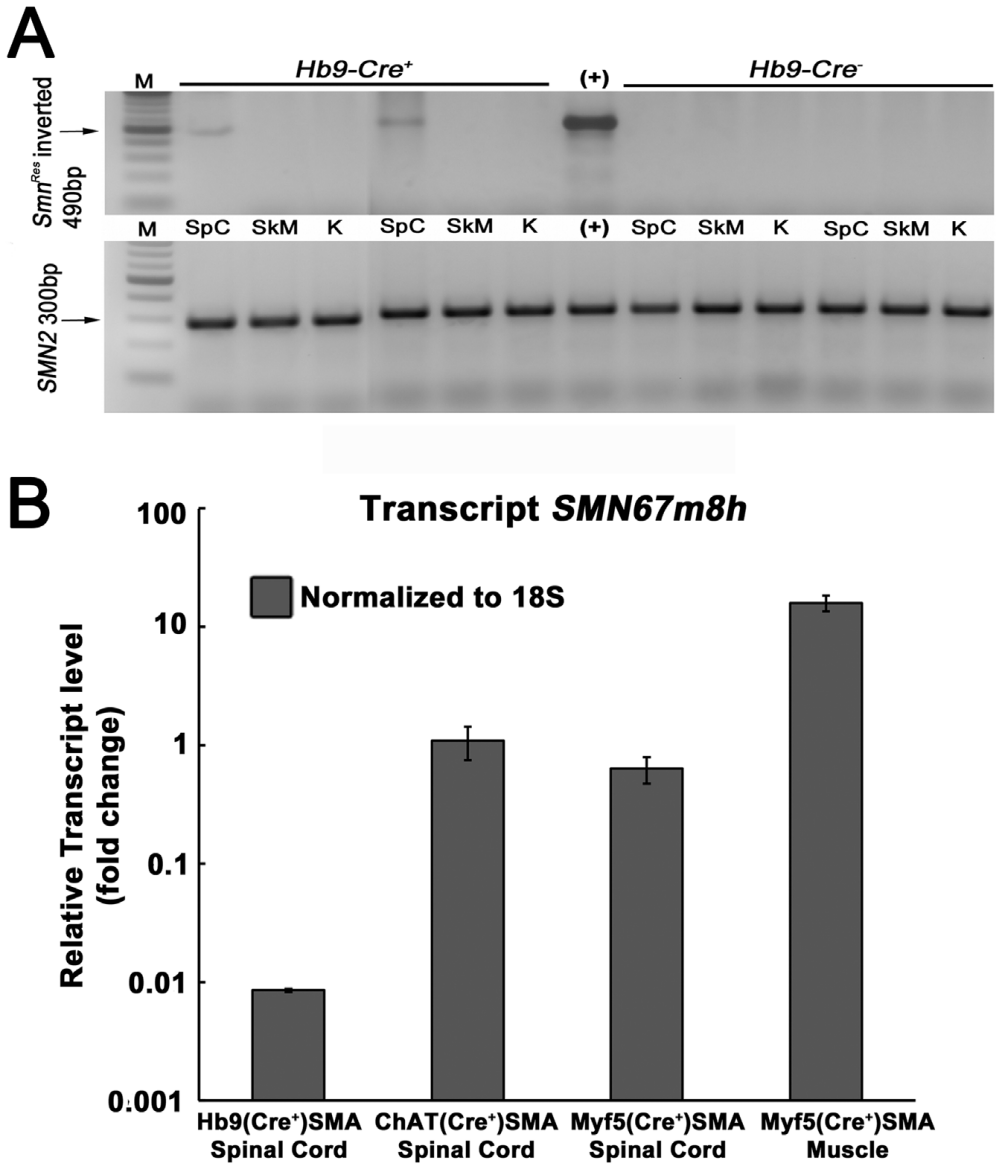
A. Spinal cord-specific Cre recombination of the *Smn^Res^* in Hb9(Cre^+^)SMA mice. Genomic DNA was prepared from spinal cord (SpC), skeletal muscle (SkM), and kidney (K) from 2 *Hb9Cre^+^* and 2 *Hb9Cre^−^* SMA animals postmortem. All animals died within the first two postnatal weeks suggesting they were *Smn^Res/Res^*. Top panels: Genomic PCR specific for the inverted, recombined *Smn^Res^* allele demonstrated its presence only in the spinal cord samples and not in the skeletal muscle or kidney samples. All these samples were positive for *SMN2*, which showed DNA integrity. Sizes of the expected DNA bands are indicated to the left of each panel. M: 100 bp ladder. (+): positive controls. Spleen DNA from a germline inverted *Smn^Res/Res^* animal was used as positive control for the recombined allele PCR. **B. Relative** ***SMN67m8h*** **expression in** ***Hb9^Cre^*** **,** ***ChAT^Cre^*** **and** ***Myf5^Cre^*** **SMA lines at P4.** The ordinate, in log scale, shows *SMN67m8h* expression normalized to levels in *ChAT^Cre^* SMA spinal cord. *Hb9^Cre^* SMA spinal cord displayed ∼100-fold lower expression than *ChAT^Cre^* SMA spinal cord. N = 2–4, per genotype.

Genomic PCR confirmed spinal cord-selective, *Hb9^Cre^* driven recombination of *Smn^Res^* in our Hb9(Cre^+^)SMA mice as the repaired allele was only detected in spinal cord, but not in skeletal muscle, kidney (Fig 2A), brain, heart or stomach (data not shown).

### Comparison between *Hb9^Cre^* and *ChAT^Cre^* driven repair of the *Smn^Res^* allele

We used quantitative RT-PCR to measure the levels of the recombined, repaired transcript *SMN67m8h*, which encodes full-length SMN (Fig 1), in P4 spinal cord and muscle. We chose this early time point because disease symptoms were not overt at this stage. We followed the same methodology described by Martinez and colleagues and used as controls P4 spinal cord and muscle tissue from their *ChAT^Cre+^* and *Myf5^Cre+^* conditional SMAΔ7 mice [13]. Primers were designed to recognize the *SMN67m8h* transcript. The forward primer cut across *Smn* exons 6 and 7 exon/intron boundary and the reverse primer bound to *SMN2* exon 8 ([13]; Fig.1). Cycle threshold (Ct) values obtained for 18S rRNA were used to equalize differences in total RNA per sample. Transcript level fold change was determined by the 2^-DeltaDeltaCt^ method [24] and values were normalized to the Ct values obtained for *ChAT^Cre+^; Smn^Res/Res^* spinal cord samples. In our hands, relative *SMN67m8h* expression levels for *ChAT^Cre+^; Smn^Res/Res^* and *Myf5^Cre+^; Smn^Res/Res^* samples were very similar to those reported before [13] (Fig 2B). In fact, the Ct values obtained with these samples were almost identical to the Ct values obtained previously with other samples with the same genotypes [13]. However, P4 *Hb9^Cre+^; Smn^Res/Res^* spinal cord had approximately 100-fold lower levels of *SMN67m8h* transcript than *ChAT^Cre+^; Smn^Res/Res^* spinal cord (Fig 2B). Consistent with our prior results at the genomic level (Fig 2A), *SMN67m8h* transcript was undetectable in muscle samples from either *Hb9^Cre+^; Smn^Res/Res^* or *Hb9^Cre-^; Smn^Res/Res^* mice (data not shown). Nor was *SMN67m8h* detected in spinal cord from *Hb9^Cre-^; Smn^Res/Res^* animals (data not shown). Thus, although these results confirm at the mRNA level the spinal cord specific recombination driven by *Hb9^Cre^* in conditional SMAΔ7 mice, they unexpectedly show that full length SMN expression in these mice is remarkably much lower than in *ChAT^Cre+^* conditional SMAΔ7 mice.

We failed to detect an increase in SMN protein levels by Western blot in spinal cord from *Hb9^Cre+^; Smn^Res/Res^* animals relative to spinal cord from *Hb9^Cre-^; Smn^Res/Res^* mice (data not shown). This is not surprising in light of the fact that this increase was not even observed when *ChAT^Cre^* drove the repair of *Smn^Res^* in conditional SMAΔ7 mice, which is probably due to the small proportion of motor neuron-derived material in lysates from whole spinal cord.

### Survival, weight progression and motor behavior in Hb9(Cre^+^)SMA mice

**Figure 3:**
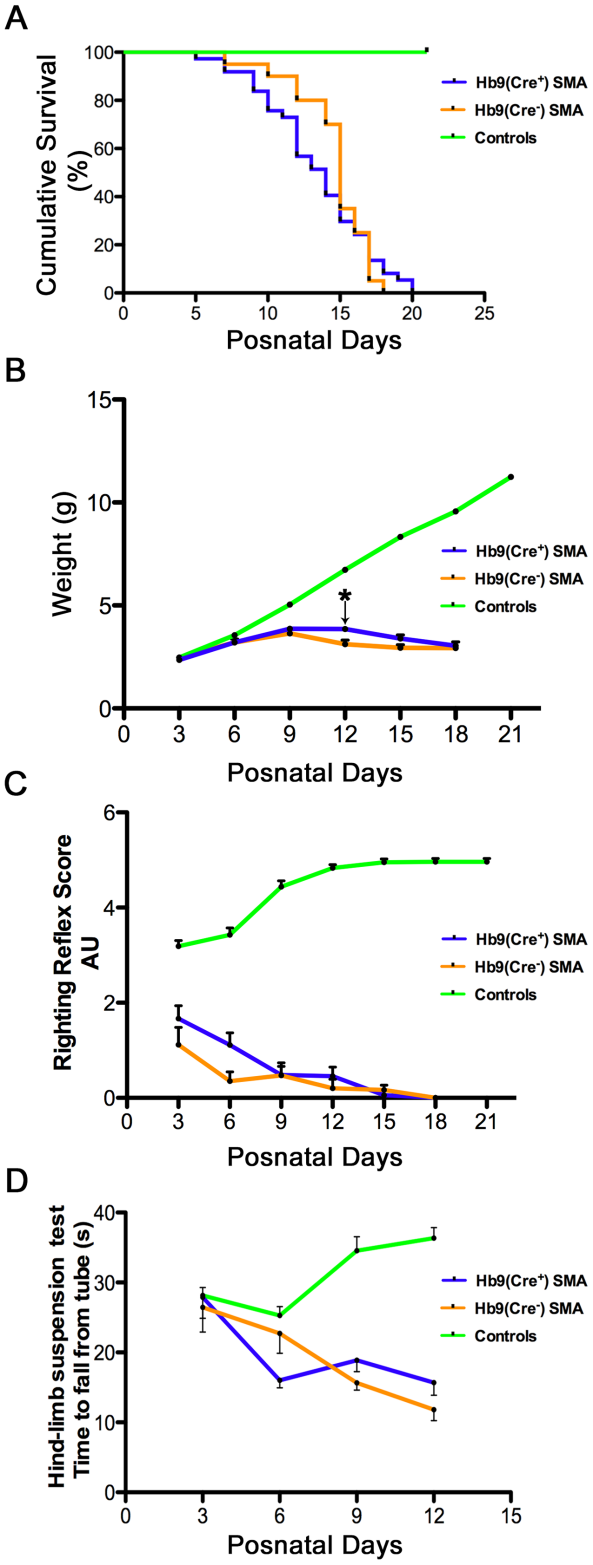
Hb9-Cre recombination fails to improve lifespan, weight gain and motor behavior in SMAΔ7 mice. **A.** Kaplan-Meier curves demonstrate no increase in survival, p = 0.6436, log-rank test. Mean life span: Hb9(Cre^+^)SMA: 13.41±3.73 days, n = 37. Hb9(Cre^−^)SMA mice: 14.6±2.64 days; n = 20. Controls: n = 130. **B**. While controls (n = 107) gained weight as expected, no statistical differences in weight were found during the lifespan of Hb9(Cre^+^)SMA and Hb9(Cre^−^)SMA mice except at P12 (*p = 0.004, F = 9.78, ANOVA). Hb9(Cre^+^)SMA: n = 10–27 per time point. Hb9(Cre^−^)SMA: n = 3–16 per time point. **C and** **D**. Motor behavior was assessed by the righting reflex (C) and hindlimb (tube) suspension (D) tests. Only the latency to fall was recorded for the tube assays. While controls improved their performance with age, SMA mice motor behavior deteriorated as they approached end stage. For the righting reflex assays: Controls: n = 107 per time point. Hb9(Cre^+^)SMA: n = 7–27 per time point. Hb9(Cre^−^)SMA: n = 4–16 per time point. For the tube test: Controls: n = 148 per time point. Hb9(Cre^+^)SMA: n = 7–28 per time point. Hb9(Cre^−^)SMA: n = 5–17 per time point.

Having obtained evidence of spinal cord-selective recombination and expression, we next characterized the phenotype of Hb9(Cre^+^)SMA mice. Median survival for Hb9(Cre^−^)SMA mice was 14.6±2.64 days (n = 20) (Fig 3A). This value agrees very well with the mean survival reported by others for these mice [11], [13] and for the original SMAΔ7 animals [4], which suggests that our breeding strategy did not alter the basic phenotype of the mice. Hb9(Cre^+^)SMA mice displayed a statistically similar lifespan (Fig 3A; 13.41±3.73 days, n = 37, p = 0.6436, log-rank test). Both SMA lines were clearly unable to gain the weight that controls did (Fig 3B). *Hb9^Cre+^* and *Hb9^Cre-^* conditional SMAΔ7 mice gained some weight between P3 and P9, but it declined steadily henceforth until time of death (Fig 3B). Hb9(Cre^+^)SMA mice tended to be a little heavier than Hb9(Cre^−^)SMA mice between P9 and P18, but this difference was statistically significant by ANOVA only at P12 (F = 9.78; p = 0.004). Motor behavior was assessed using the righting reflex and the hindlimb suspension tests (Fig 3C and 3D). As expected heterozygous and WT controls performed much better than SMA mice in these tests. No statistical differences in motor function were apparent between Hb9(Cre^+^)SMA and Hb9(Cre^−^)SMA mice. Thus we failed to detect improvements in lifespan and motor behavior in Hb9(Cre^+^)SMA animals, even though there was a slight tendency for increased weight.

### Neuromuscular phenotypes in Hb9(Cre^+^)SMA mice

**Figure 4:**
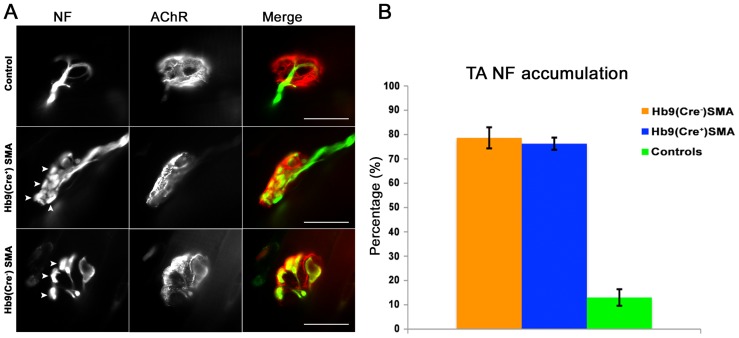
Neurofilament (NF) accumulation at nerve terminals fails to be rescued in Hb9(Cre^+^) SMA mice. Whole mounts of P10 TA muscle stained for components of the NMJ. **A**. Representative images for each of the 3 genotypes assayed. Protuberances or “blebbing” of the nerve terminals (arrowheads) are distinct evidence of SMA pathology. Green: NF, Red: BTX. Scale bars: 20 µm. **B**. Quantification of NF accumulation as seen in A. The ordinate plots the mean percentage of NMJs with NF accumulation per genotype. Most NMJs in Hb9(Cre^+^) and Hb9(Cre^−^) SMA mice had NF accumulations, while only a minority of endplates in controls showed so. Controls n = 9 animals, 107 NMJs. Hb9(Cre^+^)SMA n = 4 animals, 52 NMJs. Hb9(Cre^−^)SMA n = 3 animals, 95 NMJs.

While Hb9-Cre-driven rescue of conditional SMAΔ7 mice had no apparent effect at the whole animal level, it was still possible that their NMJ pathology could be rescued by this approach. Accumulations of neurofilament (NF) staining at the nerve terminal are a hallmark pathological feature in SMA mice [5], [6], [8], [28], and they were reduced to control levels in *ChAT^Cre+^* conditional SMAΔ7 mice [13]. We stained whole mount preparations from P10 tibialis anterior (TA) muscle for NF and AChRs to mark NMJs. While about 10% of endplates had pre-synaptic NF accumulations in controls, over 80% of NMJs displayed them in Hb9Cre+ or Hb9Cre- conditional SMAΔ7 preparations (Fig 4). Thus, NF accumulations in nerve terminals persisted in Hb9(Cre^+^)SMA mice.

**Figure 5:**
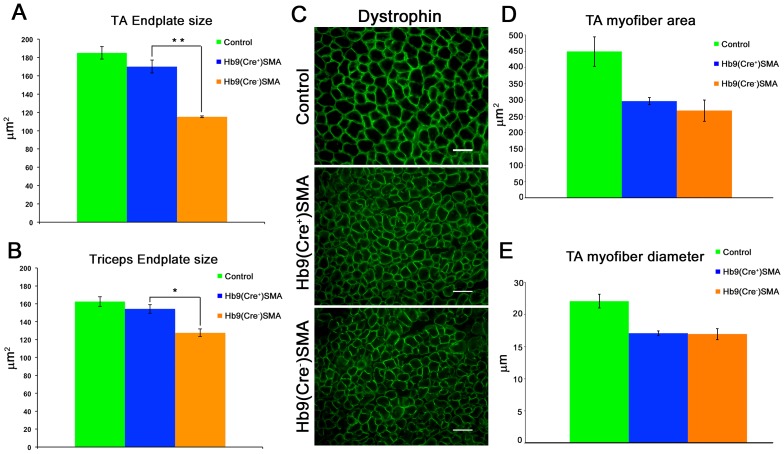
Endplate size is rescued in Hb9(Cre+) SMA mice without change in myofiber size. ** A. and B.** Quantification of endplate size in P9 TA and triceps muscles, respectively. TA endplate size was determined in whole-mount preparations. Triceps endplate area was determined in longitudinal sections from images such as that displayed in Fig 6A. Endplate area was rescued in both muscles in Hb9(Cre^+^)SMA mice. TA: Controls: n = 5 animals, 512 NMJs. Hb9(Cre^+^)SMA n = 3 animals, 465 NMJs. Hb9(Cre^−^)SMA n = 3 animals, 303 NMJs. Triceps: Controls n = 5 animals, 505 endplates. Hb9(Cre^+^)SMA n = 3 animals, 524 endplates. Hb9(Cre^−^)SMA n = 3 animals, 316 endplates. **p = 0.002. *p = 0.008 (t-test). **C**. Representative images of dystrophin stain for 14- µm-thick transverse sections of control and SMA P9 TA muscles. Dystrophin staining is qualitatively similar between genotypes but differences in fiber caliber between control and SMA muscles are evident. Scale bars: 50 µm. **D. and E**. Quantification of mean myofiber area and diameter of P9 TA muscles, respectively. Controls: n = 2 animals, 3116 fibers. Hb9(Cre^+^)SMA n = 3 animals, 5729 fibers. Hb9(Cre^−^)SMA n = 3 animals, 4511 fibers.

Endplate size in TA muscle, as measured by the area occupied by AChRs, was one of the synaptic phenotypes rescued in *ChAT^Cre+^* conditional SMAΔ7 mice [13]. We determined AChR area in whole mount TA preparations and in longitudinal sections of triceps muscles from P9 animals. To capture many NMJs per image and to ensure imagining of the entire junctional surface, wide field 3D images of the BTX-stained tissue were taken with an epifluorescence microscope fitted with a motorized Z-axis. Using Methamorph software, the Z-stacks were first flattened to the X/Y dimensions and endplate area was determined after thresholding of images. The average TA endplate areas for control (185.10±6.87 µm^2^) and Hb9(Cre^−^)SMA (115±0.94 µm^2^) genotypes were very similar to those reported previously for P9 TAs in control and SMAΔ7 mice, respectively, which were imaged by confocal microscopy [6]. In Hb9(Cre^+^)SMA mice average TA endplate area was 170.11±7.08 µm^2^. Figure 5A,B shows that endplate area for both TA and triceps NMJs was restored to control values in Hb9(Cre^+^)SMA mice. Thus, appendicular muscles innervated by pools of motor neurons in opposite ends of the spinal cord, cervical (triceps), lumbar/sacral (TA), had their average endplate area rescued in Hb9(Cre^+^)SMA mice. In normal animals endplate size correlates with myofiber size [29]. Following labeling of transverse sections for dystrophin to mark cell surface, we determined mean myofiber area and diameter in TA muscles from P9 controls, Hb9(Cre^+^) and Hb9(Cre^−^)SMA mice. We focused on this muscle because it was used previously by Martinez et al [13] in their analysis of these parameters in *ChAT^Cre^*-, *Myf5^Cre^*- and *MyoD^iCre^*- SMAΔ7 mice. Dystrophin staining was qualitatively similar between genotypes (Fig 5C). As expected, mean myofiber area and diameter were larger in controls than in SMA mice and were within the range reported previously [6], [13]. However, there were no differences in these parameters between Hb9(Cre^+^) and Hb9(Cre^−^)SMA mice (Fig 5D,E) (Control, area = 449.4±44.94 µm^2^; diameter = 22.07±1.08 µm. Hb9(Cre^+^)SMA, area = 297.06±11.10 µm^2^; diameter = 17.53±0.37 µm. Hb9(Cre^−^)SMA, area = 268.05±32.49 µm^2^; diameter = 16.96±0.86 µm). Thus, the increase in endplate size in the TA muscle from Hb9(Cre^+^)SMA mice was observed in the absence of a corresponding increase in myofiber area and diameter.

**Figure 6:**
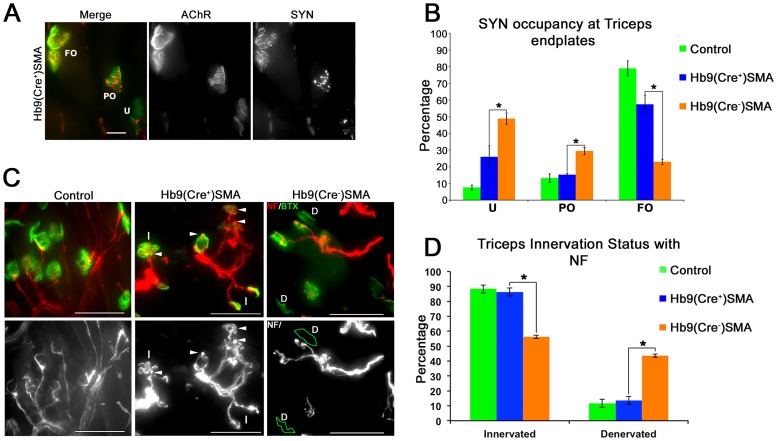
Innervation status is improved in Hb9(Cre^+^) SMA mice. **A.** A longitudinal section (40 µm-thick) from triceps muscle, from a P9 Hb9(Cre^+^)SMA animal, co-stained for synaptophysin (SYN) and BTX, to label nerve terminals and AChRs, respectively. A flattened 2D image derived from a wide field 3D stack for the fluorescein (AChR) and rhodamine (SYN) channels and their merge is shown. Two NMJs in the upper left have complete coverage of SYN staining over AChR staining, and are examples of fully-occupied (FO) NMJs. Two endplates in the bottom right (arrow), one sideways, one en face, lack detectable SYN staining, and are examples of unoccupied (U) endplates. The one endplate in the middle is partly covered by SYN staining and is an example of a partially-occupied (PO) NMJ. Scale bar: 20 µm. **B**. Quantification of synaptophysin occupancy of triceps endplates. The percentage of fully-occupied NMJs was increased, and the percentage of partially- and unoccupied endplates were reduced in Hb9(Cre^+^)SMA mice relative to Hb9(Cre^−^)SMA animals (*p<0.008, t-test). Controls: n = 5 animals, 403 endplates. Hb9(Cre^+^)SMA: 3 animals, 394 endplates. Hb9(Cre^−^)SMA: 3 animals, 439 endplates **C**. Top panels: Representative merged images of P9 triceps longitudinal sections stained for NF (red) and BTX (green). Bottom panels: NF channel in black & white. In the top left panel (control) axons can be seen overlapping part of the BTX stain or barely entering the synaptic site (endplate on the lower right corner). Arrowheads in the middle panels indicate NF accumulations at synaptic sites on Hb9(Cre^+^) SMA tissue. All the synapses in the middle panels were considered as innervated, three of them are indicated as (I). The right top panel (Hb9 (Cre^−^) SMA) shows two examples of endplates completely lacking NF/BTX overlap and thus considered denervated (D). These two endplates have been outlined in green in the bottom right panel, to further clarify their lack of overlapping NF staining. Scale bars: 50 µm. **D**. Quantification of triceps innervation using NF/BTX overlap as criterion. The percentage of innervated endplates was increased and the percentage of denervated NMJs were reduced to control levels in Hb9(Cre^+^)SMA mice relative to Hb9(Cre^−^)SMA animals (*p = 0.0004, t-test). Controls: n = 3 animals, 533 endplates. Hb9(Cre^+^)SMA: 3 animals, 478 endplates. Hb9(Cre^−^)SMA: 3 animals, 559 endplates.

Ko and colleagues demonstrated a selective vulnerability to denervation of specific, clinically relevant muscles in SMAΔ7 mice [9]. Denervation of vulnerable muscles was restored to control values in *ChAT^Cre+^* conditional SMAΔ7 mice [13]. We determined the innervation status of NMJs in the triceps, one of the reported vulnerable appendicular muscles [9], by first co-staining longitudinal sections for AChRs and synaptophysin, a synaptic vesicle marker. While lack of synaptophysin staining may not be indicative of structural denervation (i.e. absence of an axon terminal), increased synaptophysin coverage of an endplate is necessarily associated with axon terminal presence. Images were taken and processed as above. Endplates were classified as fully-occupied if at least ∼75% of the AChR area was covered by synaptophysin staining. On the other hand, they were deemed as unoccupied if less than ∼25% of the AChR area showed synaptophysin staining. Endplates with synaptophysin staining in between were categorized as partially occupied. We found a statistically significant improvement in the synaptophysin coverage of the P9 triceps endplates in Hb9(Cre^+^)SMA mice (Fig 6B). Thus, the fraction of fully-occupied synapses increased in *Hb9^Cre+^* SMAΔ7 mice over that in SMA mice [Hb9(Cre^+^)SMA = 57.47±5.58%; Hb9(Cre^−^)SMA = 23.00±1.65%], while the fraction of unoccupied endplates decreased in the rescued animals relative to SMA mice [Hb9(Cre^+^)SMA = 25.97±6.2%; Hb9(Cre^−^)SMA = 48.93±3.37%]. Partially-occupied synapses also were reduced in numbers in Hb9(Cre^+^)SMA mice [15.29±0.86% vs. 29.46±2.11% in Hb9(Cre^−^)SMA animals]. However, the improvement in synaptophysin occupancy in the *Hb9^Cre+^* SMAΔ7 mice did not quite reach the levels observed in the control animals (Fig 6B). To directly assess structural innervation, we co-stained serial triceps longitudinal sections for AChRs and neurofilament, a structural axonal marker. Synaptic sites labeled with BTX were considered innervated if there was overlap, however small, between NF and AChR staining, while complete lack of NF at synaptic sites categorized them as denervated (Fig 6C). Innervation was also improved clearly in Hb9(Cre^+^)SMA triceps using overlap between NF and BTX as scoring criterion (Fig 6D). Thus, Hb9(Cre^−^)SMA triceps had 56.29±1.11% of innervated endplates, whereas Hb9(Cre^+^)SMA triceps displayed 86.36±2.6% innervated synapses, a percentage similar to controls.

Thus, of the three NMJ structural phenotypes examined, endplate area and innervation status, but not presynaptic NF accumulation, showed significant improvements in Hb9(Cre^+^)SMA mice relative to diseased animals. Interestingly the increase in endplate size appeared to occur in the absence of an increase in myofiber size.

## Discussion

Compared to previous studies [13], [14], [30] ours seems to be the one in which restored spinal cord SMN levels are the lowest. Yet we were able to document full rescue of endplate size regardless of the susceptibility of a muscle to denervation and substantial recovery of innervation on a denervation vulnerable muscle. The increase in endplate size was not the result of an increase in muscle fiber size. The implication for therapeutics evaluation of this observation is that rescue of endplate size could be interpreted as a direct benefit on motor neurons rather than an indirect result of an increase in muscle fiber girth. Hence, our experiments uniquely highlight the exquisite sensitivity of these NMJ phenotypes to very small changes in SMN in spinal cords. These two phenotypes can be rescued by SMN levels that fail at reducing presynaptic NF accumulation, increasing animal weight and lifespan or improving overall motor performance. All of the above phenotypes were rescued or improved in *ChAT^Cre+^* conditional SMAΔ7 mice [13], which our data suggest display substantially more efficient Cre-mediated recombination in spinal cord than *Hb9^Cre+^* conditional SMAΔ7 mice. Despite the inefficient restoration of motoneuronal SMN in *Hb9^Cre+^* conditional SMAΔ7 mice, the subclinical neuromuscular innervation improvements that can be measured in them raise the possibility that similar benefits could be seen in SMA patients even when the latter are under therapeutic interventions with known modest effects in raising SMN levels.

The pattern of structural neuromuscular phenotypes rescued in *Hb9^Cre+^* conditional SMAΔ7 mice was very similar to that in *Myf5^Cre+^* conditional SMAΔ7 mice. Thus, TA endplate size and innervation status of a vulnerable muscle (the splenius capitis), but not levels of NF accumulation, were rescued in *Myf5^Cre+^* conditional SMAΔ7 mice. *Myf5^Cre+^* is strongly expressed in skeletal muscle but also weakly in spinal cord, including motor neurons (Fig 2B, [13]). Although whole spinal cord samples from *Myf5^Cre+^* and *ChAT^Cre+^* conditional SMAΔ7 mice seemed to have similar *SMN67m8h* levels (Fig 2B, [13]), it is important to point out that only a fraction of the full-length SMN expression in *Myf5^Cre+^* conditional SMAΔ7 mice may come from motor neurons themselves as *Myf5^Cre+^* was found strongly expressed in ChAT-negative ventral horn neurons and in dorsal root ganglia [13]. Thus, it is possible that expression of *Myf5^Cre+^* and *Hb9^Cre+^* in motor neurons may be more similar than suggested by our qRT-PCR in whole spinal cord samples. Our results with *Hb9^Cre+^* SMA mice strongly suggest that the rescue of endplate size and innervation status in *Myf5^Cre+^* SMA mice is explained by its expression in motor neurons and not in muscle. This is consistent with the observation that these phenotypes were not rescued in *MyoD^iCre+^*-expressing animals, which only had Cre expression in muscle but not spinal cord [13]. In normal animals, there is a direct correlation between muscle fiber girth and endplate size [29]. However, TA fiber diameter increased in *MyoD^iCre+^*-expressing animals without a corresponding increase in endplate size [13] and the increase in P9 TA endplate size observed here occurred without a change in average muscle fiber size (Fig 5C-E). Thus, together these data suggest that endplate size, at least in SMA severe mice, is largely a motor neuron cell autonomous phenotype. While structural NMJ phenotypes, excluding NF accumulation, were improved in *Hb9^Cre+^*- and *Myf5^Cre+^*-conditional SMAΔ7 mice (this work and [13]), central structural phenotypes (i.e. loss of motor neuron numbers and of glutamatergic synapses onto their somas), which we did not analyze here, were not rescued in *Myf5^Cre+^*-expressing animals [13]. They were mitigated in the higher SMN expressing *ChAT^Cre+^* conditional SMAΔ7 mice [13]. Together the above results suggest that peripheral structural phenotypes may be more sensitive to levels of SMN than central structural phenotypes.

The expression patterns for *ChAT* and *Hb9* largely overlap in that they both are present in motor neurons. *Hb9* may come up earlier during development than *ChAT*, but it is not surprising that the latter drives higher levels of transcription than the former, as transcription factor genes are generally expressed at lower level than other genes. Sets of non-overlapping interneurons in the spinal cord also express *Hb9* and *ChAT*. The former is expressed in glutamatergic V_x_ interneurons, while the latter is expressed in cholinergic V0_c_ interneurons [27]. V0_c_ interneurons are marked by expression of the Pitx2 transcription factor and interestingly modulate motor output amplitude [31]. It is a remote, though testable, possibility that restoration of SMN expression in V0_c_ interneurons in *ChAT^Cre^* ^+^ conditional SMAΔ7 mice contributes to the difference between our results and those of Martinez and colleagues [13]. Much more likely is the possibility that recombination of the *Smn^Res^* allele is more efficiently driven by *ChAT^Cre^* than by *Hb9^Cre^* simply because higher levels of the recombinase result from the former than the latter. Thus, one possibility is that recombination fails in many more motor neurons in Hb9(Cre^+^)SMA than in ChAT(Cre^+^) SMA mice. This may be critical to mitigate whole animal phenotypes such as survival, weight and motor function but not NMJ phenotypes such as endplate size and innervation status as long as the endplates where these phenotypes are examined are innervated by motor neurons where Cre recombination occurred. Another possibility is that both *Smn^Res^* alleles are repaired in more *ChAT^Cre^*-expressing than *Hb9^Cre^*-expressing motor neurons. This would translate into higher levels of SMN protein per motor neuron in *ChAT^Cre^* animals. Thus, a combination of these non-exclusive alternatives may account for the higher *SMN67m8h* transcript levels in spinal cord from ChAT(Cre^+^)SMA mice, which lead to a more extensive rescue.

While our finding that spinal cord from *Hb9^Cre+^* conditional SMAΔ7 mice had remarkably much lower levels of full-length SMN transcript than spinal cord from *ChAT^Cre+^* conditional SMAΔ7 mice explains our results vis-à-vis those of Martinez and colleagues [13], it is more difficult to account for the results of Gogliotti and colleagues [14], who used the same *Hb9^Cre^* line we used here, albeit to rescue a different conditional *Smn* allele (*Smn^2B-Neo^*). To generate *Smn^2B-Neo^*, three nucleotide substitutions (GGA→TTT) were engineered in the exon splice enhancer within *Smn* exon 7 and a *pgk-neo* cassette, flanked by WT *loxP* sites, was inserted downstream in the intron between *Smn* exons 7 and 8 [12]. Upon Cre recombination, *the pgk-neo* cassette is excised, and the allele becomes *Smn^2B^* [12], [32]. *Smn^2B/2B^* mice show no signs of SMA phenotype and have a normal lifespan, despite ∼66% decrease in SMN protein in spinal cord [32]. Contrary to *Smn^Res^* [11], the *Smn^2B-Neo^* allele yields transcripts encoding full-length SMN prior to Cre recombination (∼7.5% WT allele levels [12]), although like *SMN2*, the majority of its transcripts encode SMNΔ7 [12]. *Smn^2B-Neo/2B-Neo^* mice are embryonic lethal that die between E9.5-E12.5, later than *Smn* ^−/−^ embryos, which die at E7.5 [12]. Full length SMN protein could be detected in *Smn^2B-Neo/2B-Neo^* embryos at 1–3% WT levels, somehow lower than predicted by the levels of transcript [12]. This embryonic lethality can be rescued in *Smn^2B-Neo/2B-Neo^*; *SMN2^+/−^* mice, which die perinatally around P7 [14]. Like us, Gogliotti et al. used *Hb9^Cre^* to selectively restore SMN in motor neurons of *Smn^2B-Neo/2B-Neo^*; *SMN2^+/−^* mice [14]. However, unlike results reported here with conditional SMAΔ7 mice, they found improvements in lifespan (to just P12 on average), weight, and motor behavior assayed by the righting reflex and hindlimb suspension tests, the same tests used here. NMJ pathology also improved in *Hb9^Cre+/−^*; *Smn^2B-Neo/2B-Neo^*; *SMN2^+/−^* mice. We propose that their more extensive rescue was due to the endogenous low levels of full length SMN protein afforded by the *Smn^2B-Neo^* allele, that together with those provided by the *Smn^2B^* allele (i.e. after recombination) yielded higher overall levels of SMN in motor neurons. This interpretation assumes that the recombination efficiency of *Smn^Res^* and *Smn^2B-Neo^* was the same in the two experiments as both used the same *Hb9^Cre^* line. However, the recombination efficiency may not only depend on the levels of Cre but also on the nature of the two conditional alleles. Hence, to be repaired *Smn^2B-Neo^* requires the excision of the *pgk-neo* cassette, while *Smn^Res^* requires the inversion of the 3′5′ *Smn* exon 7 (Fig 1). The *pgk-neo* cassette in *Smn^2B-Neo^* is flanked by WT *loxP* sites, while the inversion cassette in *Smn^Res^* is flanked by mutant *lox66/lox71* sites, which ensures the irreversibility of the inversion reaction [33]. To reach comparable levels of recombination efficiency in vitro, Cre-mediated inversion of *lox66/lox71*-flanked DNA regions requires 2–3 fold more Cre than Cre-mediated excision of WT *loxP*-flanked DNA regions [33]. In vivo, the same study showed that *lox66/lox71* inversion was slightly less efficient than WT *loxP* excision [33]. Thus, it is possible that the *Hb9^Cre^*-driven recombination efficiency of *Smn^2B-Neo^* is higher than that for *Smn^Res^*. This, together with the likely higher basal levels of full-length SMN in *Smn^2B-Neo/2B-Neo^*; *SMN2^+/−^* mice relative to conditional SMAΔ7 mice, may account for the amelioration of less SMN-sensitive phenotypes observed by Gogliotti et al. [14], which we failed to observe in our experiments. Recent experiments by others indicate that pathology in cells other than motor neurons critically underlie the short lifespan of severe SMA mice (for review see [34]). As the two lines carrying the inducible conditional *Smn* alleles, *Smn^Res^* and *Smn^2B-Neo^*, continue to be used to determine the contribution of other cell types to the SMA phenotype and lifespan, it will be important that the research community be aware of the differences between these two conditional lines regarding their endogenous SMN levels and their differential demand for Cre drivers. While used successfully by many for excision of floxed alleles (e.g. references in http://jaxmice.jax.org/strain/006600.html), our results raise the possibility that *Hb9^Cre^* might not be a robust Cre driver for inversion of floxed alleles in general.

Notwithstanding the involvement of other cell types suggested by animal models, survival and other defects in motor neurons are still expected to be hallmark features of the human disease. Having biomarkers that directly reflect changes in motor neuron phenotypes following a therapeutic approach is still very important. The apparent higher SMN sensitivity and the easier experimental accessibility of the NMJ better position peripheral structural phenotypes to serve as biomarkers for testing therapeutic interventions than central structural phenotypes. Indeed, Ko and colleagues already demonstrated that denervation of vulnerable muscles can serve as a very sensitive biomarker to evaluate pharmacological interventions in SMAΔ7 mice [9]. Results of the present study further support this conclusion. Our analysis of endplate area in one denervation-vulnerable (triceps) and one denervation-resistant (TA) muscle, innervated by motor neuron pools located in opposite ends of the spinal cord, indicates that this phenotype could also be used as a sensitive motor neuron-autonomous marker to evaluate drug therapies in SMAΔ7 mice. Although both parameters appear equally sensitive to small increases in SMN levels, endplate size has the potential advantage of applying to all muscles, regardless of their vulnerability to denervation.
